# Association of adverse childhood experiences with functional identity and impulsivity among adults: a cross-sectional study

**DOI:** 10.12688/f1000research.13007.2

**Published:** 2018-05-18

**Authors:** Muhammad Haaris Sheikh, Sadiq Naveed, Ahmed Waqas, Ihtisham Tahir Jaura

**Affiliations:** 1 King Edward Medical University, Lahore, Pakistan; 2 KVC Prarie Ridge Psychiatric Hospital, Kansas City, USA; 3 CMH Lahore Medical College & Institute of Dentistry, Lahore Cantt, Pakistan

**Keywords:** adverse childhood, impulsivity, identity, Pakistan, abuse

## Abstract

**Introduction**: The present study explores the association of adverse childhood experiences with impulsivity and functional identity among Pakistani adults.

**Methods**: In this cross-sectional study, 260 Pakistani medical students aged 18 and above were approached. A consent form, a questionnaire on sociodemographic characteristics, and an English versions of the Adverse Childhood Experiences (ACE) scale, Functions of Identity scale (FIS) and Barratt’s Impulsiveness Scale (BIS-11) was employed in this study. All data were analyzed in SPSS v. 20.

**Results**: A total of 122 (52.6%) of respondents had experienced at least one adverse childhood experience. Verbal, physical, sexual adverse events and poor support and affection from family were the most reported adverse events. ACE scores yielded a significantly positive association with cognitive stability, perseverance and motor impulsivity on the Barrat’s impulsivity scale. Whereas, it yielded negative association with structure and harmony subscales of the functional identity as well as cognitive complexity subscale of the impulsivity scale.

**Conclusions**: A high proportion of Pakistani medical students reported adverse childhood experiences, which lead to impulsive behaviors and poor functional identities.

## Introduction

The Centers for Disease Control and Prevention (CDC) report a high prevalence of physical (28%) and sexual abuse (21%) associated with an unstable living environment among the American youth ^1^. Previous studies demonstrate a significant relationship between experience of abuse and physical, behavioral and social problems among the youth ^1^. Although there is abundant data exploring the prevalence of adverse childhood experiences in higher income countries, in low and middle income countries (LAMI) data is more scarce ^2^ Moreover, a paucity of data has been identified in the LAMI, necessitating the need to transculturally translate the impact of adverse childhood events (ACEs) on social, cognitive and emotional impairment and adoption of high risk behaviors ^3^.

Childhood emotional mistreatment; particularly emotionally abusive acts, has been found to be associated with increased odds of lifetime diagnoses of several mental disorders ^4^. The early, prolonged, and severe trauma can also increase impulsivity, diminishing the capacity of the brain to regulate emotions. Neurobiological studies show that childhood mistreatment leads to failure of inhibitory processes ruled by the frontal cortex over a fear-motivated hyper-responsive limbic system ^5^. Therefore, impulsivity is a double edged sword, presenting itself as sequela of trauma as well as a risk factor for the development of a pathological response to trauma ^6^. Many psychiatric disorders feature impulsivity, including substance-abuse disorders, attention deficit hyperactivity disorder, borderline personality disorder, conduct disorder and mood disorders. Impulsivity has also been associated with suicidal behaviors within various psychiatric populations exhibiting low serotonergic activity ^7^. In mental health disorders especially substance use disorders, superimposition of the behavioral aftermaths of ACEs on impulsivity potentiate the risk of alcohol abuse by many folds ^8^.

Similarly, previous studies have also established an association between ACEs and development of identity in adolescence. Development of a stable identity is a major developmental task, with its changing facets responsible for shaping the attachment styles and self-esteem in adolescence ^9, 10^. Serafini and Adams describe the importance of identity in providing structure for higher self-esteem and positive self-image; providing the goals necessary for self-direction ^11^. This provides a sense of free will; harmony for social and academic adjustment; and future orientation that manifests as achievements in academia, aspirations and determination ^11^. To address the gaps in scientific literature, the present study explores the association of adverse childhood experiences with demographics, subsequent impulsivity and functional identity among Pakistani adults.

## Methods

This study was designed as a cross-sectional study, where 260 medical students aged 18 and above and currently enrolled in King Edward Medical University and CMH Lahore Medical College & Institute of Dentistry, both in Lahore, were conveniently interviewed from April to May, 2017. Institutional review board approval was sought and obtained from the Ethical Review Board of CMH Lahore Medical College, Pakistan (approval number: 21/ERC/CMHLMC). A consent form, an anonymous questionnaire on sociodemographic characteristics, and English versions of the Adverse Childhood Experiences (ACE) scale, Functions of Identity scale (FIS) and Barratt’s Impulsiveness Scale (BIS-11) were employed in this study. Participation in this study was voluntary and written informed consent was obtained from all participants. The participants were ensured anonymity and that only group findings would be reported.

The Adverse Childhood Experiences (ACE) questionnaire is an important assessment tool that measures multiple types of abuse and adverse experiences that one may have encountered as a child ^1^. It assesses adverse childhood experiences related to abuse (physical, psychological and sexual); neglect (emotional and physical) and household dysfunction (alcoholism or drug use at home, loss of biological parent, mental illness in home, violent treatment by mother and imprisoned household member). Responses to the ACE are recorded on a dichotomous scale (yes/no) and then scores are summed with higher scores corresponding to a higher number of ACEs. It has exhibited adequate reliability (Cronbach’s alpha 0.6 to 0.8) and validity in previous study ^1^.

The Functions of Identity Scale (FIS) is a valid and reliable 5-point Likert scale, comprising 15 questions that assess five domains of psychological functions that identity serves for an individual: structure, goals, personal control, harmony and future ^11^. Higher scores on these subscales correspond to a stronger sense of identity.

Barratt’s Impulsiveness Scale (BIS-11) is a 30-item self-report Likert scale, with seven subscales; attention, motor, self-control, cognitive complexity, perseverance, and cognitive instability ^12^. Higher scores on the scale or its subscales correspond to worsening impulsivity. All of these scales were found to be reliable in the present sample with following Cronbach’s α; ACE (0.71), FIS (0.86) and BIS-11 (0.78).

All data were analyzed in SPSS v. 21. Descriptive statistics were computed for the whole data. Frequencies were calculated and reported for ten domains of ACE, impulsivity and functions of identity. Partial correlations were run to assess the association of impulsivity and functions of identity with ACEs, adjusting for gender, age and socioeconomic status.

## Results

A total of 232 medical students (232/260= 89.2%) responded to the surveys. The majority of them were females (n=188, 81%), with a mean age of 21.22 ± 1.31 years, mean number of siblings 3 ± 1.46, mean order of birth 1.94 ± 0.78 and a mean income greater than 30,000 PKR (n=208, 89.7%). Mean scores on subscales of Functional Identity Scale and Barratt’s Impulsiveness Scale are given in Table 1.

**Table 1.** Mean scores on subscales of the Functional Identity Scale.

Subscale	Mean	Std. Deviation
Functional Identity Scale		
Structure	11.14	2.5
Harmony	12.27	2.3
Goals	11.59	2.6
Future	11.00	3.0
Personal Control	11.78	2.1
Barrat’s Impulsiveness Scale		
Attention	11.46	2.9
Cognitive instability	7.47	2.1
Motor	16.50	3.9
Perseverance	7.57	2.0
Self-control	13.13	3.4
Cognitive complexity	12.14	2.6
Attention	18.93	3.9
Motor	24.08	4.8
Non-planning	25.27	4.9

Mean score (SD) on the ACE scale was 1.37 (1.75). A total of 122 (52.6%) respondents had experienced at least one ACE. Verbal, physical, sexual adverse events and poor support and affection from family were the most reported adverse events. A significant proportion of respondents cited verbal (34.5%), physical (22.0%) and sexual abuse (15.5%), poor family support (19.0%), neglect (9.9%), separation/divorce of parents (4.7%), and witnessed domestic abuse (11.2%), substance abuse (3.9%), mentally or suicidal patient in the family (11.2%) and criminal background (4.7%). Detailed statistics are presented in Table 2.

**Table 2.** Adverse childhood experiences reported by respondents.

Adverse childhood experiences	Response	Count	Column N %
Did a parent or other adult in the household often? Swear at you, insult you, put you down, or humiliate you? or Act in a way that made you afraid that you might be physically hurt?	No	152	65.5%
	Yes	80	34.5%
Did a parent or other adult in the household often: Push, grab, slap, or throw something at you? or Ever hit you so hard that you had marks or were injured?	No	181	78.0%
	Yes	51	22.0%
Did an adult or person at least 5 years older than you ever: Touch or fondle you or have you touch their body in a sexual way? or Try to or actually have oral, anal, or vaginal sex with you?	No	196	84.5%
	Yes	36	15.5%
Did you often feel that no one in your family loved you or thought you were important or special? or Your family didn’t look out for each other, feel close to each other, or support each other?	No	188	81.0%
	Yes	44	19.0%
Did you often feel that you didn’t have enough to eat, had to wear dirty clothes, and had no one to protect you? or Your parents were too drunk or high to take care of you or take you to the doctor if you needed it?	No	209	90.1%
	Yes	23	9.9%
Were your parents ever separated or divorced	No	221	95.3%
	Yes	11	4.7%
Was your mother or stepmother: Often pushed, grabbed, slapped, or had something thrown at her? or Sometimes or often kicked, bitten, hit with a fist, or hit with something hard? or Ever repeatedly hit over at least a few minutes, or threatened?	No	206	88.8%
	Yes	26	11.2%
Did you live with anyone who was a problem drinker or alcoholic or who used street drugs	No	223	96.1%
	Yes	9	3.9%
Was a household member depressed or mentally ill or did a household member attempt suicide?	No	206	88.8%
	Yes	26	11.2%
Did a household member go to prison?	No	221	95.3%
	Yes	11	4.7%

ACE scores yielded a significantly positive association with cognitive stability, perseverance and motor impulsivity on the Barrat’s impulsivity scale. Whereas, it yielded negative association with structure and harmony subscales of the functional identity as well as cognitive complexity subscale of the impulsivity scale. Detailed statistics are presented in Table 3. Moreover, no significant correlation was found with gender (P= 0.07), number of siblings (P= 0.95) and order in birth (P=0.08) and hoursehold income (P= 0.21). Age of participants was positively associated with ACE scores (r= 0.15, P= 0.02).

**Table 3.** Association of ACE scores with subscales of impulsivity and functional identity (n=223).

Variable	r *	P-value
Impulsivity		
Attention	0.038	0.575
Cognitive stability	0.133	0.046
Perseverance	0.145	0.029
Self-control	0.008	0.901
Cog complx	-0.227	0.001
Attention	0.101	0.130
Motor	0.151	0.024
Non-planning	-0.115	0.085
Functional identity		
Structure	-0.219	0.001
Harmony	-0.169	0.011
Goals	-0.012	0.855
Future	0.005	0.941
Personal control	-0.060	0.374

*Controlled for gender, age, year of study, number of siblings and order in birth

Impulsivity and adverse childhood events The dataset contains all variables pertaining to demographics, responses to Functional Identity Scale and Barrat’s Impulsiveness Scale. Click here for additional data file. Copyright: © 2018 Haaris Sheikh M et al. 2018 Data associated with the article are available under the terms of the Creative Commons Zero "No rights reserved" data waiver (CC0 1.0 Public domain dedication).

## Conclusion

In our study, adverse childhood experiences were significantly negatively associated with structure and harmony subscales of the functional identity scale. Providing structure is a major function of one’s identity, deprivation of this results in poor self-esteem and negative self-image ^11^. These adverse experiences may provide a better orientation in adulthood to fulfill one’s potential in academics and career in adulthood ^11^.

Individuals reporting higher episodes of ACEs reported higher impulsivity, translating to a greater motor impulsiveness and a disrupted executive functioning among these individuals ^12^.

The results of this study should be generalized with caution. The cross-sectional nature of this study does not establish causality and temporality, therefore, future studies should employ a longitudinal study design.

## Data availability

The data referenced by this article are under copyright with the following copyright statement: Copyright: © 2018 Haaris Sheikh M et al.

Data associated with the article are available under the terms of the Creative Commons Zero "No rights reserved" data waiver (CC0 1.0 Public domain dedication). 

**Dataset 1: Impulsivity and adverse childhood events.** The dataset contains all variables pertaining to demographics, responses to Functional Identity Scale and Barrat’s Impulsiveness Scale. DOI, 10.5256/f1000research.13007.d182670 ^13^.

## Consent

Participation in this study was voluntary and written informed consent was obtained from all participants. The participants were ensured anonymity and that only group findings would be reported.
