# Supplementary material for: Association of adverse childhood experiences with functional identity and impulsivity among adults: a cross-sectional study
Source: F1000Res. 2018 May 18;6:1978. Originally published 2017 Nov 8. [Version 2] doi: 10.12688/f1000research.13007.2 (PMC6117857; doi:10.12688/f1000research.13007.2)
Supplement: Peer review history

# Peer review: Referee response for version 2

**DOI:** 10.5256/f1000research.16311.r35983

**Published:** 2018-08-29

**Authors:** Muhammad Shahzeb Khan

**Affiliations:** 1 Department of Internal Medicine, John H. Stroger, Jr. Hospital of Cook County, Chicago, IL, USA

The article addresses an important issue especially among the Pakistani Population. Considering its a short report, the authors have done a good job in their literature review. However as I am not an expert in this field, cannot comment on the technicalities and intrinsic details of the subject matter. The methods and analysis seem appropriate. In the abstract results section, it will be better to provide numbers, percentages where they talk about frequency of adverse events. In the abstract methods, they should state which analysis they ran.

I have read this submission. I believe that I have an appropriate level of expertise to confirm that it is of an acceptable scientific standard.

# Peer review: Referee response for version 2

**DOI:** 10.5256/f1000research.16311.r34188

**Published:** 2018-07-02

**Authors:** Syeda Fariha Iram Rizvi

**Affiliations:** 1 Govt, M A O College, Lahore, Pakistan

The article is improved, but still I have one problem - you have used one nominal scale with yes/no and two are ordinal scales. In this case you can't compute Pearson correlation. I think you have to use chi square and logistic regression. I would suggest to consult any statistics expert.

I have read this submission. I believe that I have an appropriate level of expertise to confirm that it is of an acceptable scientific standard, however I have significant reservations, as outlined above.

# Response

**Published:** 2018-07-29

**Authors:** Ahmed Waqas

**Affiliations:** CMH Lahore Medical College, Pakistan

Dear Dr. Rizvi,

                        My co-authors and I are very grateful to you for your feedback. We had used total scores on the Adverse Childhood Experiences (ACE) scale, Functions of Identity scale (FIS) and Barratt’s Impulsiveness Scale (BIS-11). Therefore, these were measured as continuous variables (scale) rather than ordinal or dichotomous. Hence, use of partial correlations is justified in this case.

 We would be grateful if you could please reconsider your decision in light of our response.

 Best wishes,

 Dr. Ahmed Waqas

 Corresponding author

# Peer review: Referee response for version 1

**DOI:** 10.5256/f1000research.14105.r31204

**Published:** 2018-03-19

**Authors:** Syeda Fariha Iram Rizvi

**Affiliations:** 1 Govt, M A O College, Lahore, Pakistan

Childhood abuse is under recognized phenomena in Pakistan. This research is good effort to link childhood adverse experiences with impulsivity and identity. but there are few confusions/questions. Some detail of ACE questionnaire should be mentioned. Explain sub-variables in ACE in terms of questions. Is every single question of ACE is a separate variable?  how we can say that if a person say yes to a single question that means he or she had a adverse childhood experience?  Only Frequency and severity of an experience can determine its intensity.

 Literature review is poor. Objectives and hypotheses are not mentioned in article.

 I have serious concerns regarding result/analysis section. Relationship should be analysed with correlational analysis first and then go for regression analysis to confirm the relation while identifying significant predictors.

 Analysis is very much confused as I can’t understand that what variables are described as predictors and which one are outcome variables. According to Table no3 impulsivity and identity are describes as predictors/independent variables although according to title and literature ACE are predictors and impulsivity and identity related variables are outcome variables. So according to the purpose of research results are wrong. If author has something else in mind please explain it.

 If author will mention objectives and hypotheses and then give analyses according to hypotheses then reader can understand what actually author want to explore.

 Discussion is poorly written. please relate your results with existing literature

I have read this submission. I believe that I have an appropriate level of expertise to state that I do not consider it to be of an acceptable scientific standard, for reasons outlined above.

# Response

**Published:** 2018-05-12

**Authors:** Ahmed Waqas

**Affiliations:** CMH Lahore Medical College, Pakistan

**Response to Reviewer:**

 Dear Ms. Rizvi,

                             We are grateful to you for providing such a valuable feedback to our short report exploring the association of adverse childhood experiences with functional identity and impulsivity among adults. We have updated our manuscript in line with your comments, and firmly believe that it has improved the quality of our manuscript.

 We hope for your favorable response in due time.

 Best wishes,

 Dr. Ahmed Waqas

 Corresponding author

 Comment 1:

 Explain sub-variables in ACE in terms of questions. Is every single question of ACE is a separate variable? 

 Response 1:

 We are grateful to you for your feedback. We have expanded the methodology section with details on ACE questionnaire. It now reads as:

 “The Adverse Childhood Experiences (ACE) questionnaire is an important assessment tool that measures multiple types of abuse and adverse experiences that one may have encountered as a child ^1^ . It assesses adverse childhood experiences related to abuse (physical, psychological and sexual); neglect (emotional and physical) and household dysfunction (alcoholism or drug use at home, loss of biological parent, mental illness in home, violent treatment by mother and imprisoned household member). Responses to the ACE are recorded on a dichotomous scale (yes/no) and then scores are summed with higher scores corresponding to a higher number of ACEs. It has exhibited adequate reliability (Cronbach’s alpha 0.6 to 0.8) and validity in previous study ^1^.”

 Comment 2:

 how we can say that if a person say yes to a single question that means he or she had a adverse childhood experience?  Only Frequency and severity of an experience can determine its intensity.

 Response 2:

 The Adverse Childhood Experiences scale is one of the most widely used scales globally. It has demonstrated adequate factor validity as well as reliability in previous studies. Based on these merits, we had opted to use this scale in our setting. Furthermore, it also yielded an acceptable reliability (alpha= 0.71) in our study.

 Please, also see:

1. Felliti V, Anda R: The Adverse Childhood Experiences (ACE) study. Atlanta, GA Center of Disease Control and Prevention.1997. Reference Source
2. Ford DC, Merrick MT, Parks SE, Breiding MJ, Gilbert LK, Edwards VJ, Dhingra SS, Barile JP, Thompson WW. Examination of the factorial structure of adverse childhood experiences and recommendations for three subscale scores. Psychology of violence. 2014 Oct;4(4):432.

 

  Comment 3:

 Literature review is poor. Objectives and hypotheses are not mentioned in article.

 Response 3:

 We partly agree with your comment. But please, do understand that our manuscript is a short report, bound by a word limit of 1000 words excluding tables and references. Due to constraints of word count, we had provided a summary of the recent literature. And therefore, could not expand the introduction section, which has a word count exceeding 1200 at present.

 We have updated our introduction with objectives of the study.

 It reads as:

 “To address the gaps in scientific literature, the present study explores the association of adverse childhood experiences with demographics, subsequent impulsivity and functional identity among Pakistani adults.”

 Comment 4:

 I have serious concerns regarding result/analysis section. Relationship should be analysed with correlational analysis first and then go for regression analysis to confirm the relation while identifying significant predictors. Analysis is very much confused as I can’t understand that what variables are described as predictors and which one are outcome variables. According to Table no3 impulsivity and identity are describes as predictors/independent variables although according to title and literature ACE are predictors and impulsivity and identity related variables are outcome variables. So according to the purpose of research results are wrong. If author has something else in mind please explain it.

 If author will mention objectives and hypotheses and then give analyses according to hypotheses then reader can understand what actually author want to explore.

 Response 4:

 Dear Ms. Rizvi, thank you so much for your guidance. We have updated our results with new analyses providing mean scores of ACE scale (representing severity of adverse experiences) and frequency of individual types of ACEs. We have also replaced regression analysis with partial correlations adjusted for gender, age, year of study, number of siblings and order in birth.

 The results section now reads:

 Mean score (SD) on the ACE scale was 1.37 (1.75). A significant proportion of respondents cited verbal abuse (34.5%), physical (22.0%), sexual (15.5%), poor family support (19.0%), neglect (9.9%), separation/divorce of parents (4.7%), and witnessed domestic abuse (11.2%), substance abuse (3.9%), mentally or suicidal patient in the family (11.2%) and criminal background (4.7%).

 ACE scores yielded a significantly positive association with cognitive stability, perseverance and motor impulsivity on the Barrat’s impulsivity scale. Whereas, it yielded negative association with structure and harmony subscales of the functional identity as well as cognitive complexity subscale of the impulsivity scale. Moreover, no significant correlation was found with gender (P= 0.07), number of siblings (P= 0.95) and order in birth (P=0.08) and hoursehold income (P= 0.21). Age of participants was positively associated with ACE scores (r= 0.15, P= 0.02).

 Comment 5:

 Discussion is poorly written. please relate your results with existing literature

 Response 5:

 We agree with your comment. But please, do understand that our manuscript is a short report, bound by a word limit of 1000 words excluding tables and references. Therefore, we have replaced our discussion section with conclusion and limitations.

# Peer review: Referee response for version 1

**DOI:** 10.5256/f1000research.14105.r28427

**Published:** 2017-12-12

**Authors:** Usman Ali

**Affiliations:** 1 Fauji Foundation Hospital, Rawalpindi, Pakistan

In Pakistan, the researchers often have to deal with scarcity of existent data on virtually any subject. This article gives insight about adverse childhood experiences and its impact in terms of functional identity and impulsivity. However, there are a few points to be addressed. 

 First, the title states, Association of adverse childhood experiences with functional identity and impulsivity among adults; a cross sectional study. However, the study population consists of medical students from early adulthood. This should be reflected in the title.

 Secondly, the religious and culturally constrained environment is different from other low and middle income countries. Hence, whenever a study in social sciences is conducted prior validity of used questionnaire should be established, which was not done in this study.

 Furthermore, in the conclusion the authors have stated a 'high' proportion of subjects who suffered from adverse childhood experiences. What were the control cut off values for high vs low proportion in this regard.

I have read this submission. I believe that I have an appropriate level of expertise to confirm that it is of an acceptable scientific standard.
